# Supplementary material for: P-TRAP: a Panicle Trait Phenotyping tool
Source: BMC Plant Biol. 2013 Aug 29;13:122. doi: 10.1186/1471-2229-13-122 (PMC3848748; doi:10.1186/1471-2229-13-122)
Supplement: Additional file 7 — P-TRAP source code. The Netbeans project that contains the source code of the application. [file 1471-2229-13-122-S7.zip › javadoc.zip!/javadoc/org-bioalg-imageprocessor/org/bioalg/imageprocessor/HSBPixel.html]

HSBPixel (Image Processor)


JavaScript is disabled on your browser.


- Package
- Class
- Tree
- Deprecated
- Index
- Help

*org.bioalg.imageprocessor 1.0*

- Prev Class
- Next Class

- Frames
- No Frames

- All Classes

- Summary:
- Nested |
- Field |
- Constr |
- Method

- Detail:
- Field |
- Constr |
- Method


org.bioalg.imageprocessor

## Class HSBPixel

- java.lang.Object
- - org.bioalg.imageprocessor.HSBPixel

- ---

    

  ```
  public class HSBPixel
  extends java.lang.Object
  ```

  The Hue Saturation Brightness model.

  Author:
  :   faroqal-tam

- - ### Constructor Summary

    Constructors

    Constructor and Description

    `HSBPixel(float h,
    float s,
    float b)`

    `HSBPixel(RGBPixel rgb)`
  - ### Method Summary

    Methods

    | Modifier and Type | Method and Description |
    | --- | --- |
    | `double` | `distance(HSBPixel hsb)` |
    | `double` | `distancehs(HSBPixel hsb)` |
    | `double` | `distances(HSBPixel hsb)` |
    | `double` | `distancesb(HSBPixel hsb)` |
    | `float` | `getB()` |
    | `float` | `getH()` |
    | `float` | `getS()` |
    | `void` | `setB(float b)` |
    | `void` | `setH(float h)` |
    | `void` | `setS(float s)` |
    | `java.lang.String` | `toString()` |

    - ### Methods inherited from class java.lang.Object

      `clone, equals, finalize, getClass, hashCode, notify, notifyAll, wait, wait, wait`

- - ### Constructor Detail


    - #### HSBPixel

      ```
      public HSBPixel(float h,
              float s,
              float b)
      ```


    - #### HSBPixel

      ```
      public HSBPixel(RGBPixel rgb)
      ```
  - ### Method Detail


    - #### getH

      ```
      public float getH()
      ```


    - #### setH

      ```
      public void setH(float h)
      ```


    - #### getS

      ```
      public float getS()
      ```


    - #### setS

      ```
      public void setS(float s)
      ```


    - #### getB

      ```
      public float getB()
      ```


    - #### setB

      ```
      public void setB(float b)
      ```


    - #### toString

      ```
      public java.lang.String toString()
      ```

      **Overrides:**
      :   `toString` in class `java.lang.Object`


    - #### distance

      ```
      public double distance(HSBPixel hsb)
      ```


    - #### distancehs

      ```
      public double distancehs(HSBPixel hsb)
      ```


    - #### distancesb

      ```
      public double distancesb(HSBPixel hsb)
      ```


    - #### distances

      ```
      public double distances(HSBPixel hsb)
      ```


- Package
- Class
- Tree
- Deprecated
- Index
- Help

*org.bioalg.imageprocessor 1.0*

- Prev Class
- Next Class

- Frames
- No Frames

- All Classes

- Summary:
- Nested |
- Field |
- Constr |
- Method

- Detail:
- Field |
- Constr |
- Method
